# Alteration of hyperpolarization-activated cation current-mediated metaplasticity contributes to electroconvulsive shock-induced learning and memory impairment in depressed rats

**DOI:** 10.3389/fpsyt.2024.1365119

**Published:** 2024-06-07

**Authors:** Li Ren, Jian Yu, Hengsheng Chen, Jie Luo, Feng Lv, Su Min

**Affiliations:** ^1^ Department of Anesthesiology, The First Affiliated Hospital of Chongqing Medical University, Chongqing, China; ^2^ Department of Psychiatry, Shanghai 10th People’s Hospital, Anesthesia and Brain Research Institute, Tongji University, Shanghai, China; ^3^ Ministry of Education Key Laboratory of Child Development and Disorders, China International Science and Technology Cooperation Base of Child Development and Critical Disorders, Chongqing Key Laboratory of Pediatrics, Chongqing, China

**Keywords:** electroconvulsive shock, depression, metaplasticity, excitability, hyperpolarization-activated cation current

## Abstract

**Background:**

Accompanied by a rapid and effective antidepressant effect, electroconvulsive shock (ECS) can also induce learning and memory impairment. Our previous research reported that metaplasticity is involved in this process. However, the mechanisms still remain unclear. This study investigated the role of *I*_h_ current in the metaplastic changes and learning and memory impairment induced by ECS in depressive rats.

**Methods:**

Depressive rats received ECS after modelling using chronic unpredictable. ZD7288, a type of *I*_h_ current inhibitor was used to verify the effect of *I*_h_ current. The sucrose preference test and Morris water maze were used for behavior testing. Changes in metaplasticity was assessed with the LTD/LTP threshold by stimulation at different frequencies. Spontaneous and evoked action potentials (APs) were measured to confirm difference of neuronal excitability. Additionally, the amplitude of *I*_h_ current was analyzed.

**Results:**

ECS exerts antidepressant effect, but also induce spatial learning and memory dysfunction. ECS up-regulates the LTD/LTP threshold. In rats treated with ECS, the frequency of spontaneous and evoked APs is significantly reduced. In addition, ECS induces changes in the intrinsic properties of AP, including a decrease of AP-half width and peak amplitude, and an increase in AP time to peak and post-hyperpolarization potential amplitude. In particular, ECS increases both instantaneous and steady-state *I*_h_ currents. However, Inhibition of *I*_h_ current with ZD7288 results in a relief of learning and memory impairment and a decrease in threshold, as well as a significant reversal of whole-cell electrophysiological changes.

**Conclusion:**

ECS-induced learning and memory impairment is caused by neuronal hypoexcitability mediated metaplasticity, and upregulation of LTD/LTP threshold by an increase in *I*_h_ current.

## Introduction

Depression is a prevalent affective disorder, with approximately 4.7% of the world’s population experiencing a depressive episode each year (1). According to the report from the Global Burden of Disease Study, the number of people suffering from depression has reached nearly 300 million (2). Among complex treatment strategies, particularly for major or drug-resistant depression, electroconvulsive therapy (ECT) remains the most effective treatment with best empirical evidence (3). However, accompanied with the excellent antidepressant efficacy, ECT may also induce learning and memory impairment, the changes have been reported to last up to 6 months or longer and may result in functional impairment (4). After the ECT course, anterograde and retrograde amnesia are impaired (5). Some patients even feel distressed with loss of autobiographical memory (6), which may affect their willingness to receive the treatment. Unfortunately, the mechanisms by which ECT induces learning and memory impairment remains poorly understood and need to be urgently investigated.

The role of synaptic plasticity in the learning and memory function is well established. And changes in synaptic efficacy, such as long-term potentiation (LTP) impairment has been proved to involve in learning and memory dysfunction in many pathological models (7, 8). In our previous study, we also found electroconvulsive shock (ECS), an analog of ECT in animals, caused LTP impairment and learning and memory dysfunction (9). Why ECS can induce LTP damage then becomes next question. Further studies have shown that synaptic plasticity may be influenced by the history of synaptic plasticity, a mechanism of synaptic homeostasis named metaplasticity (10). The modification of synaptic strength by metaplasticity depends mainly on the adjustment of the LTD/LTP threshold (11). An increase in threshold can suppress subsequent LTP and facilitate LTD. Alternatively, when threshold is reduced, synapses are more willing to promote LTP and inhibit LTD. In our further study, it was found that ECS-induced learning and memory impairment was metamorphosed by an increase in threshold (12). However, the mechanisms underlying ECS-induced threshold increase in metamorphosis are not fully understood.

It is well known that changes in neuronal excitability are involved in synaptic transmission and synaptic plasticity. There is growing evidences that neuronal excitability is enhanced or diminished following activity deprivation or hyperactivity of neural circuits to maintain neural homeostasis (13, 14). Crestani and his colleagues also found that prior learning could increase neuronal excitability, which serves as a metaplastic mechanism for subsequent memory formation (15). It is now thought that neuronal excitability is importantly correlated with learning and memory through a metaplastic mechanism that regulates threshold (16). The hyperpolarization-activated cationic current (*I*_h_) is an inward mixed cation current consisting of Na^+^ and K^+^, that contribute greatly to the neuronal excitability. Reducing *I*_h_ has been reported to increase the intrinsic excitability of hippocampal neurons (17). While artificial increasing *I*_h_ leaded to a reduction of firing rating in response to current injection (18). Therefore, *I*_h_ is thought to be a mediator for the threshold sliding within metaplasticity framework (19). Nevertheless, it remains unclear whether ECS-induced cognitive dysfunction and metaplasticity regulation are attributed to changes in neuronal excitability and *I*_h_.

In this study, an electrophysiological technique was conducted to investigate whether ECS induces changes in *I*_h_ current, neuronal excitability and metaplasticity in depressive rats. Further, we used ZD7288, a selective *I*_h_ inhibitor, to verify whether ECS-induced cognitive dysfunction was caused by alteration of neuronal excitability-mediated metaplasticity by increased *I*_h_.

## Materials and methods

### Animal

Male Sprague-Dawley rats, weighing 200-250g and aged 2-3 months, were obtained from the Laboratory Animal Center of Chongqing Medical University. All rats were housed at standardized laboratory conditions (temperature of 22 ± 2°C, humidity of 62% ± 3% and 12/12 h light-dark cycle). All experiment protocols were approved by the Ethical Committee of the First Affiliated Hospital of Chongqing Medical University and were performed in accordance with the National Institutes of Heathy Guild for the Care and Use of Laboratory Animals.

### Depressive model constructed with chronic unpredictable mild stress

All the procedures were conducted as previously published method, with minor modification (20). Rats were housed individually in cages and randomized to two stressors per day for 28 days: food deprivation for 24 h, water deprivation for 24 h, tailing pinching for 1 min, continuous lighting for 24 h, 4°C cold water swim for 5 min, 45°C hot water swim for 5 min, shaking for 20 min, damp sawdust for 24 h, cage tilting to 45°C from horizontal for 24h. Anhedonia is defined as one of the core symptoms of depression and can be detected by sucrose preference test As described in previous reports (21, 22), rats with a sucrose preference percentage (SPP) <65% were identified as rats with depression-like behavior rats and were screened for the following experiments.

### Sucrose preference test

This test was conducted as previously described (23). Briefly, all rats were required to adapt to sucrose consumption prior to the formal experiment. After 23 h of water and food deprivation, rats were provided with two identical bottles (one with 1% sucrose solution and one containing water) to drink freely for 1 h, swapping the positions of the two bottles at half the test time. The sucrose preference percentage was calculated as the formula: SPP (%) =sucrose consumption (g)/[water consumption (g) +sucrose consumption (g)] × 100%.

### Morris water maze

Morris water maze is widely used to evaluate spatial learning and memory functions in rats. In our experiment, this test was conducted the day after the completion of ECS or sham ECS treatment. In brief, a circle pool with a submerge platform was artificially divided into four quadrants. Rats were placed gentle from four different quadrants into the water to find the platform. The time to find the platform was calculated and defined as the escape latency If the rats could not find the platform within 60 s, they were guided to platform and the time was recorded as 60 s. The experiment lasts for 5 consecutive days. On the sixth day, the platform was removed and rats were placed from four different quadrants into the water and swam freely for 60 seconds and the time spent exploring the space was recorded and defined as the time spent swimming in the platform quadrant. In addition, the swimming speed of the rats was analyzed to exclude those with motor dysfunction.

### Groups and treatment

All the depressive rats were randomly divided into four groups: depression+sham ECS group (group Sham), depression+sham ECS+ ZD7288 (group Sham+ZD7288), depression+ECS group (group ECS), depression+ECS+ZD7288 (group ECS+ZD7288). Rats in group Sham received sham ECS and rats in group ECS received ECS. Previous studies reported that lateral ventricle injection of 5μg/kg ZD7288 does not affect locomotor activity (24, 25), so rats in group ECS+ZD7288 received 5μg/kg ZD7288 injection through lateral ventricle catheter, and then subjected to ECS 15 min later. Rats in group Sham+ZD7288 received a 5μg/kg ZD7288 injection through a lateral ventricle catheter, and then subjected to sham ECS 15 min later. ECS was performed with bilateral ear clip electrodes through a Niviqure ECS system (Nivique Meditech, Bangalore, India) with the following parameters: bidirectional square wave pulses, 0.8 A in amplitude, 1.5 ms in width, 125 Hz in frequency, 0.8 s in duration, and 120 mc in charge. Sham ECS was conducted as the same procedure with ECS, but without currents. The sham ECS and ECS treatments were conducted once a day for 7 days. These parameters and number of treatment days were determined on previous studies by our research group and others from the literature (26, 27).

### Lateral ventricular catheter surgery

Due to the impermeability of ZD7288 to the blood-brain barrier, lateral ventricular injection was adopted in this experiment. Rats were anesthetized with 2% pentobarbital, then the skull was exposed and immobilized in a stereotaxic instrument. Access to lateral ventricular was established with a drill according to coordinates as previously described: AP -1.2mm, ML +1.8mm, DV -4.5mm (28). A sterile cannula was fixed in the position with dental cement and obstructed with a stainless lock. After the surgery, the rats were kept in separate cages and gentamicin was used to prevent infection.

### Hippocampal field potential recording and LTD/LTP threshold assessment

After anesthesia with pentobarbital sodium, the brains of the rats were removed. Hippocampal slices of 400 μm were cut with a vibrating slicer (NVSLM-1, WPI, USA) in a cutting solution at 0°C-4°C under perfusion with a gas mixture of 95% O_2_ and 5% CO_2_. The solution was formulated as 3 KCl, 1.25 NaH_2_PO_4_, 26 NaHCO_3_, 0.4 vitamin C, 2 sodium pyruvate, 2 sodium lactate, 10 glucose, 220 sucrose, 2 MgCl_2_, 0.1 CaCl_2_, 4 MgSO_4_ (in mM). The slices were transferred into a chamber and incubated with oxygenated recording solution at 34°C for 60 min, then the temperature was adjusted to 24°C kept for 60 min. The recording solution was comprised of 124 NaCl, 3 KCl, 1.25 NaH_2_PO_4_, 26 NaHCO_3_, 0.4 vitamin C, 2 sodium pyruvate, 2 sodium lactate, 10 glucose, 2 CaCl_2_, 1.2 MgSO_4_ (in mM).

After incubation, the sections were fixed in the chamber with artificial nylon nets mesh and filled with oxygenated recording fluid. Stimulating electrode was placed in the stratum radiatum of the CA3 region and recording electrode (a glass micropipette with a resistance 2-3 MΩ) with filtrated recording solution was placed in the stratum radiatum of CA1 region. The stimulus intensity was set to an intensity that elicited half of maximum response. Firstly, baseline field excitatory postsynaptic potentials (fEPSPs) were recorded for 30 min, then the protocol of the same number pulses (400 pulses) with different frequencies was conducted to explore the LTD/LTP threshold crossover point, the fEPSP of post-stimulation at different frequencies were recorded for 60 min. To assess the magnitude of synaptic transmission, the mean value for the slope of fEPSPs recorded at 20-40 min after stimulation was calculated and expressed as a percentage of the mean value of the initial baseline slope of fEPSPs. The point was defined as the stimulus intensity that evoked a change in synaptic depression to potentiation. To reduce sacrifice in rats, the stimulation frequencies of the crossover point were set according to our previous report (12), and the whole frequency-response curve was not tracked in this experiment.

### Whole-cell patch-clamp electrophysiological recording

For the whole-cell recording, the procedure for section preparation was similar to that for field potential recordings, except for the formula of the artificial cerebrospinal fluid (ACSF). Sections were cut in ice-cold NMDG ACSF bubbled with the same O_2_/CO_2_ gas mixture in a solution formulated as 93 NMDG, 2.5 KCl, 1.2 NaH_2_PO_4_, 30 NaHCO_3_, 20 HEPES, 25 glucose, 5 sodium ascorbate, 2 thiourea, 3 sodium pyruvate, 12 NAC, 10 MgSO_4,_ 0.5 CaCl_2_ (in mM), and the PH adjusted to 7.3-7.4 with HCl. Sections were then incubated for 60 min at 34°C in an oxygenated modified HEPES ACSF with a culture solution consisting of 92 NaCl, 2.5 KCl, 1.2 NaH_2_PO_4_, 30 NaHCO_3_, 20 HEPES, 25 glucose, 5 sodium ascorbate, 2 thiourea, 3 sodium pyruvate, 12 NAC, 10 MgSO_4,_ 0.5 CaCl_2_ (in mM), and the PH was set to 7.3-7.4 by NaOH.

The slices were perfused continuously in the recording chamber with oxygenated ACSF containing (in mM): 124 NaCl, 2.5 KCl, 1.2 NaH_2_PO_4_, 24 NaHCO_3_, 5 HEPES, 12.5 glucose, 2 MgSO_4,_ 2 CaCl_2_, and the PH maintained at 7.3-7.4 with NaOH or HCl as necessary. Whole-cell patch-clamp recordings were made from CA1 pyramidal neurons by infrared visual video with a ×40 water immersion objective. The electrodes were made with a resistance of 4-6 MΩ and filled with pipette solution containing (in mM): 120 K-gluconate, 20 KCl, 10 HEPES, 0.5 EGTA, 2 MgCl_2_, 2 Na_2_ATP, and the PH was set to 7.3-7.4 by KOH. GΩ resistance sealing was formed between the neuron and pipette, and then the whole-cell recording was achieved by an impulsive suction. The series resistance was monitored during the recording and cells with less than 20 MΩ and in which the resistance varied by less than 20% were included. Spontaneous action potentials (APs) were recorded under current-clamp conditions in the “I=0” model and AP frequencies with a period of 20 s was calculated. Evoked APs were also examined in this experiment, and spike activity was recorded for depolarizing current (200 pA, 650 ms). Similarly, the frequencies of evoked APs were compared. It is well accepted that neuronal excitability is influenced by the active neuronal membrane properties, which is determined by changes in voltage-gated ion channels (29). And AP waveform parameters, such as AP-half width and AP peak, AP time to peak and after hyperpolarization potential (AHP) could reflect the changes of voltage-gated ion channels and neuronal excitability in the neurons. Thus, all the parameters were examined and compared in this experiment. In voltage-clamp condition, *I*_h_ current was activated by a series of voltage steps from -120 mV to -60 mV, with steps of 10 mV (1 s duration) and a holding potential of -50 mV. Instantaneous *I*_h_ current was identified as the activated current following each test potential immediately, and steady-state *I*_h_ current was identified as the current at the end of the potential. Amplitude of instantaneous and steady-state *I*_h_ current in response to -120 mV and -90 mV potentials were measured. For all patch clamp recordings, data were recorded and analyzed with Multiclamp 700B amplififier, Digidata 1400 transverter and Pclamp 9.2 software.

### Statistical analysis

All the data were expressed as mean ± SD and analyzed with Graphpad prism software (v.9.1.10). All data, except for escape latency, were analyzed by a two-way ANOVA with ECS and ZD7288 as independent variables. In the experiment of escape latency, time was regarded as another independent variable, so a three-way ANOVA was used to calculate the difference in escape latency and swimming speed between groups. *Post-hoc* Tukey’s test was used for multiple comparisons. *P*<0.05 was considered to be statistically significant.

## Results

### Alteration of rats’ depressive behavior, spatial learning and memory function

**Figure 1:**
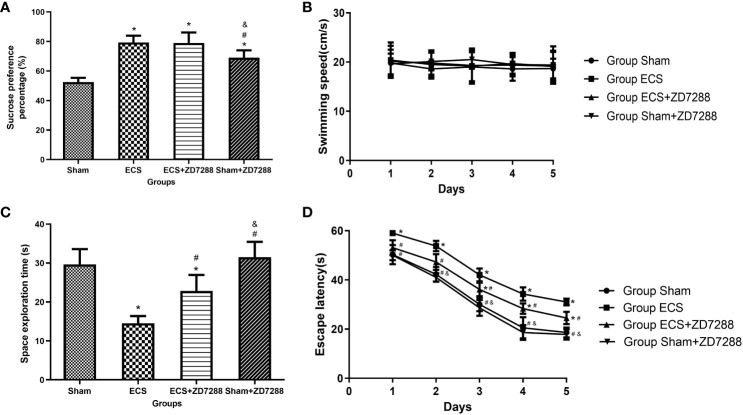
Effects of ECS and ZD7288 on behavior changes. **(A)** Results of sucrose preference test. **(B–D)** Results of Morris water maze of swimming speed, escape latency and space exploration time. Data of sucrose preference test and space exploration time were analyzed with a two-way ANOVA, swimming speed and escape latency were analyzed with a three-way ANOVA. And multiple pairwise comparisons were used for the *post hoc* tests. *P<0.05 compared with group Sham; # *P*<0.05 compared with group ECS; & *P*<0.05 compared with group ECS+ZD7288.

The experiment schedule was exhibited in the **Supplementary Figure 1**. There were six rats in each group for behavioral testing. The analysis results of SPP are shown in **Figure 1A**. Significant results were reported in the analysis of the main effect of ECS (F=60.06, *P*<0.0001) and ZD7288 (F=12.27, *P*=0.0022). Furthermore, there was a significant interaction effect between ECS and ZD7288 (F=12.27, *P*=0.0022). For multiple comparison, the SPP in the ECS, ECS+ZD7288 and Sham+ECS groups was significantly higher than that of the Sham group (*P*<0.0001, *P*<0.0001, *P*=0.0005, respectively). In contrast, the SPP in the Sham+ZD7288 group was lower than that in the ECS and ECS+ZD7288 groups (*P*=0.0415, *P*=0.0415, respectively). However, there was no difference between group the ECS and ECS+ZD7288 groups in the comparison of SPP (*P*>0.9999). Morris water maze test was applied to examine spatial learning and memory in rats in this experiment. Swimming speed was not affected by ECS treatment or ZD7288 injection, the main effect of time (F=0.2858, *P*=0.8864), ZD7288 (F=0.5135, *P*=0.4819), and ECS (F=0.1033, *P*=0.7513) were not significant, and there was no significant interaction effect between those three factors. And there was no difference in swimming speed between the groups (*P*>0.9999)(**Figure 1B**). After two-day acclimation, the results from 3^rd^ to 5^th^ days were compared and a three-way ANOVA was tested. The main effect of time (F=766.2, *P*<0.0001), ZD7288 (F=24.45, *P*<0.0001) and ECS (F=155.6, *P*<0.0001) were all significant. The interaction effect of time and ECS (F=5.012, *P*=0.0012), ZD7288 and ECS (F=11.57, *P*=0.0028) was significant. However, the interaction effect of time and ZD7288 (F=0.1318, *P*=0.9703), time, ZD7288 and ECS (F=0.1176, *P*=0.9759) was not significant. In the 3^rd^ day, rats receiving ECS treatment (group ECS and ECS+ZD7288) revealed longer time to find the platform compared to group Sham (*P*<0.0001, *P*=0.0224, respectively), additionally, the time in group ECS+ZD7288 was shorter than that in group ECS (*P*=0.0430). However, no difference was found between the Sham and Sham+ZD7288 groups in terms of escape latency (*P*>0.9999). A similar trend was found in the results on days 4 and 5 (**Figure 1D**). In the experiment of space exploration, the main effect of ECS (F=66.26, *P*<0.0001) and ZD7288 (F=12.06, *P*=0.0024) were significant. And positive results were also found in the interaction effect of ECS and ZD7288 (F=4.929, *P*=0.0381). As shown in **Figure 1C**, rats in group ECS spent the shortest time swimming in the platform quadrant in the four groups (*P*<0.0001, *P*=0.0034, *P*<0.0001, respectively), while similar time was found between in group Sham and Sham+ZD7288 (*P*=0.8123). Additionally, group Sham+ZD7288 shown longer time than group ECS+ZD7288 (*P*=0.0024).

### Metaplastic changes along with LTD/LTP threshold modification

**Figure 2:**
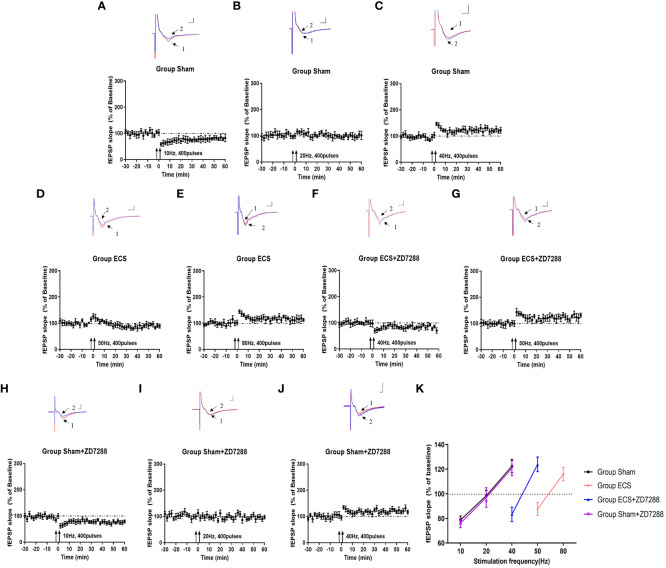
Key points with transition from synaptic depression to potentiation. Changes of synaptic transmission with different frequency stimulation in group Sham **(A–C)**, group ECS **(D–F)**, group ECS+ZD7288 **(G, H)** and group Sham+ZD7288 **(H–J)**. For the results from one specific frequency stimulation, representative traces of fEPSPs recorded in hippocampal slices were exhibited in the upper site. Trace 1 (red trace) and trace 2(blue trace) indicated the baseline and post-stimulation trace, respectively. Scale bar: 0.5mV for the vertical line and 3 ms for the horizontal line. Changes of normalized of fEPSP slopes with the stimulation were exhibited in the bottom site. **(K)** The changes in fEPSP slope on the frequency of stimulation.

In this experiment of field potential recording, hippocampal slices were counted and more than 8 slices were analyzed in each group. As shown in **Figures 2A–C**, group Sham shown synaptic depression at 10 Hz (normalized fEPSP slope, 78.7 ± 3.3%, n=8) and synaptic enhancement at 40 Hz (normalized fEPSP slope, 122.5 ± 5.4%, n=8), whereas stimulation at 20 Hz elicited neither synaptic depression nor enhancement (normalized fEPSP slope, 98.5 ± 4.3%, n=8). Results in group ECS revealed that the threshold was between 50 Hz and 80 Hz because of synaptic depression at 50 Hz (normalized fEPSP slope, 87.7 ± 5.3%, n=8) and a lower degree of potentiation at 80 Hz (normalized fEPSP slope, 116.0 ± 5.4%, n=8) (**Figures 2D, E**). With reference to group ECS, group ECS+ZD7288 exhibited lower threshold with synaptic depression at 40 Hz (normalized fEPSP slope, 83.3 ± 5.9%, n=11) and synaptic enhancement at 50 Hz (normalized fEPSP slope, 123.9 ± 5.9%, n=9) (**Figures 2F, G**). However, no effect of ZD7288 on threshold was found in depressed rats without ECS, with synaptic depression and enhancement found at 10 Hz and 40 Hz (normalized fEPSP slope, 76.3 ± 3.8%, n=10, 120.8 ± 6.1%, n=11), and no depression or enhancement was showed at 20 Hz (normalized fEPSP slope, 97.0 ± 8.0%, n=10) (**Figures 2H–J**). The results obtained with the range of different frequencies were shown in **Figure 2K**. All the results suggested that the LTD/LTP threshold was around at 20 Hz in group Sham and Sham+ZD7288, 50-80 Hz in group Sham+ZD7288 and 40-50 Hz in group ECS+ZD7288, respectively.

### Alteration of neuronal excitability evidenced by changes of spontaneous and evoked APs

**Figure 3:**
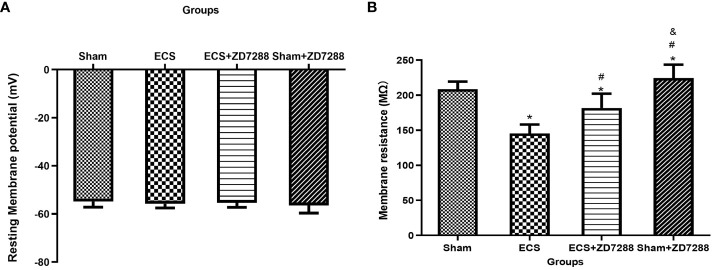
ECS and ZD7288 induced alterations in the resting membrane potential **(A)** and membrane resistance **(B)** of CA1 pyramidal neurons. *P<0.05 compared with group Sham; # *P*<0.05 compared with group ECS; & *P*<0.05 compared with group ECS+ZD7288.

**Figure 4:**
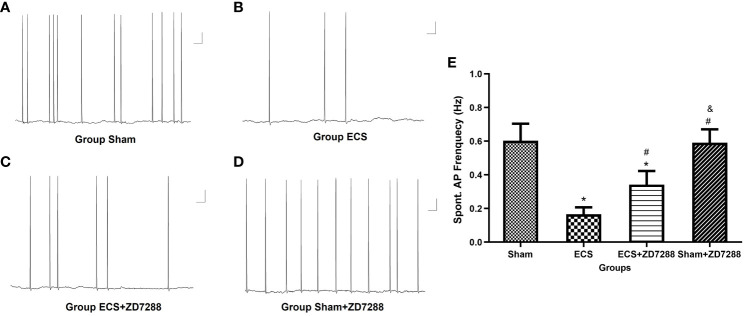
Changes of spontaneous action potential (AP) frequency of hippocampal CA1 pyramidal neurons. **(A–D)** Representative traces of spontaneous AP. Scale bar: 10 mV for the vertical line and 1 ms for the horizontal line. **(E)** Statistical analysis of the frequency of spontaneous AP in different groups. Data were analyzed with a two-way ANOVA. And multiple pairwise comparisons were used for the post hoc tests. *P<0.05 compared with group Sham; # *P*<0.05 compared with group ECS; & *P*<0.05 compared with group ECS+ZD7288.

Hippocampal CA1 pyramidal neurons were counted in the experiment of whole-cell recording. Difference in the electrophysiological properties of CA1 pyramidal neurons were observed. For membrane resistance, the main effect of ECS (F=168.1, *P*<0.0001), ZD7288 (F=40.44, *P*<0.0001) and the interaction effect (F=6.163 *P*=0.0158)were all significant. Membrane resistance significantly decreased in group ECS (145.58 ± 12.70 MΩ, n=15) compared to group Sham (208.72 ± 10.77 MΩ, n=16, *P*<0.0001). The resistance increased in group ECS+ZD7288 (181.71 ± 20.52 MΩ, n=18, *P*<0.0001) compared to group ECS. In addition, the membrane resistance in group Sham+ZD7288 (224.56 ± 19.12 MΩ, n=17, *P* =0.0464) was higher compare to group Sham. However, no significant main effect of ECS (F=0.0043, *P*=9474), ZD7288 (F=0.9368, *P*=0.3369) and the interaction effect (F=3.154, *P*=0.0807) were exhibited in reference to resting membrane potential, and no significant difference in the *post hoc* multiple comparisons (**Figures 3A, B**). For spontaneous firing frequency, the main effect of ECS (F=308.7, *P*<0.0001) and ZD7288 (F=17.44, *P*<0.0001) was significant, and the interaction effect of ECS and ZD7288 was also significant (F=22.92, *P*<0.0001). As shown in **Figures 4A–E**, in group ECS (0.16 ± 0.04 Hz, n=15) and ECS+ZD7288 (0.34 ± 0.08 Hz, n=18), the neurons exhibited a significant decrease in the spontaneous firing frequency as compared to group Sham (0.60 ± 0.10 Hz, n=16, *P*<0.0001, *P*<0.0001, respectively). However, no significant difference was found between group Sham and Sham+ZD7288 (0.59 ± 0.07 Hz, n=17, *P*=0.9986). Spontaneous frequencies in group ECS+ZD7288 and Sham+ZD2788 were higher than group ECS (*P*<0.0001, *P*<0.0001, respectively). In addition, there was also a significant difference between group ECS+ZD7288 and Sham+ZD7288 (*P*<0.0001).

**Figure 5:**
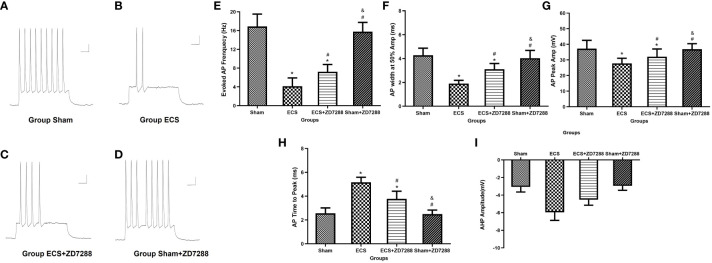
Influence of ECS and ZD7288 on the trequency and intrinsic properties of evoked action potential (AP) of hippocampal CA1 pyramidal neurons. **(A–D)** Representative traces of AP in response to depolarizing current (200 pA, 650 ms). Scale bar: 10 mV for the vertical line and 0.05 ms for the horizontal line. **(E)** Statistical analysis of the frequency of evoked AP in different groups. Statistical analysis of intrinsic properties of AP, including AP-half width **(F)**, AP time to peak **(G)**, AP peak **(H)** and after hyperpolarization potential amplitude **(I)**. Data were analyzed with a two-way ANOVA. And multiple pairwise comparisons were used for the post hoc tests. *P<0.05 compared with group Sham; # *P*<0.05 compared with group ECS; & *P*<0.05 compared with group ECS+ZD7288.

For firing frequency, the main effect of ECS was significant (F=458.3, *P*<0.0001), the interaction effect of these two interventions was also significant (F=17.84, *P*<0.0001). However, the main effect of ZD7288 was not significant (F=3.960, *P*=0.0510). CA1 pyramidal neurons in group ECS (4.13 ± 1.76 Hz, n=15) showed a strong decrease in firing frequency in response to depolarizing current injection as compared to group Sham (16.87 ± 2.62 Hz, n=16, *P*<0.0001). And ECS with ZD7288 (group ECS+ZD7288, 7.22 ± 1.55 Hz, n=18) could increase the frequency compared to group ECS (*P*=0.0003). However, no difference was found between group Sham and Sham+ZD7288 (15.76 ± 1.98 Hz, n=17, *P*=0.5314). In addition, we analyzed differences in AP waveform parameters to further reflect changes in neuronal excitability. For all the parameters, the main effect of ECS and ZD7288, also the interaction effect of these two interventions were all significant. Compared to group Sham, APs in group ECS exhibited a significant decrease in AP-half width (4.27 ± 0.59 ms, n=16 vs. 1.89 ± 0.27 ms, n=15, *P*<0.0001) and AP peak (37.22 ± 5.34 mV, n=16 vs. 27.76 ± 3.32 mV, n=15, *P*<0.0001), an increase in AP time to peak (2.55 ± 0.45 ms, n=16 vs. 5.15 ± 0.43 ms, n=15, *P*<0.0001) and AHP amplitude (-3.07 ± 0.57 mV, n=16 vs. 5.95 ± 0.91 mV, n=15, *P*<0.0001). Similar trends were found between group Sham and ECS+ZD7288 in the comparison of AP-half width (3.10 ± 0.47 ms, n=18, *P*<0.0001), AP peak (32.09 ± 4.92 mV, n=18, *P* =0.0074), AP time to peak (3.77 ± 0.64 ms, n=18, *P*<0.0001) and AHP amplitude (-4.52 ± 0.61 mV, n=18, *P*<0.0001). Furthermore, group ECS+ZD7288 exhibited a longer AP-half width (*P*<0.0001), higher AP peak (*P*=0.0385), shorter AP time to peak (*P*<0.0001) and lower AHP amplitude (*P*<0.0001) as compared to group ECS. However, the AP-half width (4.03 ± 0.64 mV, n=17, *P*=0.7029), AP peak (36.85 ± 3.58 mV, n=17, *P*>0.9999), AP time to peak (2.49 ± 0.33 ms, n=17, *P*=0.9993) and AHP amplitude (-2.94 ± 0.50 mV, n=17, *P*=0.9954) did not show differences between group Sham and Sham+ZD7288 in the comparison (**Figures 5A–I**).

### Alteration of *I*_h_ current amplitude

**Figure 6:**
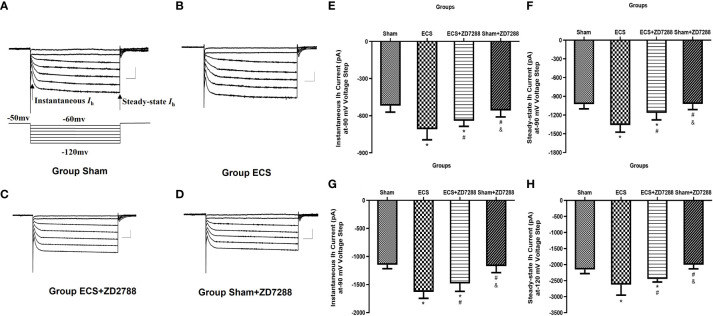
Alteration of hyperpolarization-activated cationic current (*I*_h_) of hippocampal CA1 pyramidal neurons. **(A–D)** Representative traces of *I*_h_ current activated by voltage step from -120 mV to -60 mV from a holding potential of -50 mV. The arrow indicated instantaneous *I*_h_ current at the start of potential and steady-state *I*_h_ current at the end of the potential. Scale bar: 500 pA for the vertical line and 100 ms for the horizontal line. **(E–H)** Statistical analysis of instantaneous and steady-state *I*_h_ current activated at -90 and -120 mV voltage step. Data were analyzed with a two-way ANOVA. And multiple pairwise comparisons were used for the *post hoc* tests. *P<0.05 compared with group Sham; # *P*<0.05 compared with group ECS; & *P*<0.05 compared with group ECS+ZD7288.

It is well known that changes in *I*_h_ currents are an important mechanism for neuronal excitability-mediated metaplasticity. At the voltage of -120 mV, in the analysis of instantaneous *I*_h_, the main effect of ECS (F=216.9, *P*<0.0001), ZD7288 (F=5.706, *P*=0.0199), and the interaction effect (F=10.47, *P*=0.0019) were all significant. However, in the analysis of steady-state *I*_h_, the interaction effect was negative (F=0.1193, *P*=0.7309), the main effect of ECS (F=106.6, *P*<0.0001) and ZD7288 (F=13.52, *P*=0.0005) were significant. As shown in **Figures 6A–H**, at the voltage of -120 mV, instantaneous and steady-state *I*_h_ current amplitude in group ECS were -1633.27 ± 112.28 pA (n=15) and -2630.22 ± 322.43 pA (n=15). These were significantly higher than those in group Sham (-1148.39 ± 69.78 pA, n=20, *P*<0.0001; -2156.17 ± 124.74 pA, n=20, *P*<0.0001). However, ZD7288 partially reversed the changes in *I*_h_ current amplitude induced by ECS (group ECS+ZD7288, -1481.42 ± 139.85 pA, n=17, *P*=0.0014; -2451.50 ± 92.21 pA, n=17, *P*=0.0036). And there was no difference between group Sham and Sham+ZD7288 (-1171.26 ± 116.00 pA, n=16, *P*=0.9265; -2008.15 ± 124.70 pA, n=16, *P*=0.0829).

At the voltage of -90 mV, in the analysis of instantaneous *I*_h_, the main effect of ECS (F=89.03, *P*<0.0001) and the interaction effect (F=13.91, *P*=0.0004) were significant. However, the main effect of ZD7288 was not significant (F=1.042, *P*=0.3112). In the analysis of steady-state *I*_h_, the main effect of ECS (F=102.9, *P*<0.0001), ZD7288 (F=18.17, *P*<0.0001) and their interaction effect (F=16.80, *P*<0.0001) were all significant. At a voltage of -90 mV, the instantaneous and steady-state *I*_h_ current amplitude were -519.20 ± 52.15 pA (n=20) and –1027.44 ± 73.34 pA (n=20) for group Sham, -709.52 ± 84.78 pA (n=15) and –1361.93 ± 111.48 pA (n=15) for group ECS, -640.85 ± 45.00 pA (n=17) and –1165.59 ± 111.63 pA (n=17) for group ECS+ZD7288, -558.35 ± 51.68 pA (n=16) and –1023.60 ± 162.42 pA (n=16) for group Sham+ZD7288. At a voltage of -90 mV, the statistical differences between these groups were similar to the comparative trend at the voltage of -120 mV.

## Discussion

In this experiment, we found that ECS increased the *I*_h_ current amplitude in parallel with a decrease in neuronal excitability, which was confirmed by a decrease in the frequency of spontaneous and evoked AP and changes in the intrinsic properties of AP (including AP-half width, AP peak, AP time to peak and AHP), leading to an up-regulation of the LTD/LTP threshold and learning and memory impairment. While ZD7288, an *I*_h_ inhibitor, partially reversed ECS-induced changes in neuronal excitability, metaplasticity and cognitive impairment. However, ZD7288 alone did not affect learning and memory function and mechanisms associated with metaplasticity. The present study demonstrates that ECS-induced learning and memory impairment is attributed to neuronal excitability-mediated metaplasticity by an increase in *I*_h_ current.

According to previous studies, an important debate about the longitudinal evolution of cognitive impairment caused by ECT has attracted attention. A meta-analysis confirmed that most cognitive impairments are temporary and many improvements occur one month after ECT (30). Some studies have even found that the memory is significantly better than before ECT (31), which is likely to be associated with the improvement of depressive illness (6). However, it has also been reported that ECT can induce long-term cognitive impairment (32). In our study, behavioral tests were conducted one day after ECS, and the short-term results showed that ECS can lead to learning and memory impairment. The changes in long-term cognitive function and metaplasticity were not tested in this study mainly because our previous study found that ECS-induced learning and memory began to improve on the third day after ECS, and the rat’s learning and memory function improved to baseline on the 14th day after ECS (33). Metaplasticity is a mechanism of synaptic homeostasis and can dynamically alter synaptic transmission according to the history of synaptic plasticity. Based on the mechanism, it is logical to speculate that as the influence of ECS gradually diminishes, synaptic strength should theoretically recover, and cognitive function would improve. This hypothesis is consistent with our previous finding. And on the on the other hand, the recovery of cognitive function also proves the truth of the metaplasticity theory in the ECS-induced model.

It is widely accepted that up-regulation of *I*_h_ is involved in the pathogenesis of depression (34, 35), and *I*_h_ inhibitor, such as ZD7288, has been proved to exert an antidepressant-like behavioral effect (36). In the present study, SPP was higher in group Shan+ZD7288 than in group Sham, which again demonstrates that ZD7288 does have an antidepressant effect. Nevertheless, this antidepressant effect was not exhibited in group ECS+ZD7288, as the SPP was not higher in group ECS+ZD7288 than in group ECS. A plausible explanation is that ECS is extremely efficacious in the treatment of depression and the ceiling effect comes into play (37, 38). As a consequence, the antidepressant effect of ZD7288 was masked by ECS. It has been reported that most depressed patients also suffer from cognitive dysfunction (39), however, despite improvements in depressive behavior in group Sham+ZD7288, the results of Morris water maze in group Sham+ZD7288 were not superior to those in group Sham. These data appear to be very consistent with the clinical fact that most depressed patients continue to have cognitive impairment even after remission of depressive symptoms (40, 41). Several researchers have proposed the idea that there is a separation between cognitive functioning and psychopathological symptoms in depressed patients (42). However, further research is needed to illuminate the relationship between depression and cognitive dysfunction.

Neuronal excitability serves as a metaplastic mechanism for learning and memory. Djebari et al. found when the excitability of CA1-CA3 synapses was increased, LTP was transformed into LTD and hippocampal-dependent cognitive function was impaired (43). In our study, we found ECS induced an up-regulation of the LTD/LTP threshold and reduction of the firing rate of spontaneous and evoked AP. Furthermore, ECS induced changes in intrinsic properties, in particular an increase in AHP, which is widely considered to be an indicator of neuronal excitability (16). However, when neuronal excitability was adjusted by ZD7288, the threshold was correspondingly lowered. In brief, these data confirm that ECS induces hypoexcitability and the changes in metaplasticity. Pro. Ueta and Kato also found similar results although the suppression of the pyramidal cell excitability induced by ECS exhibited a layer-specific manner (44). However, it must be stressed that hypoexcitability is exhibited after ECS. The mechanism leading to subsequent neural hypoexcitability during ECS should be spotlighted. In general, ECS is an intensive electrical stimulation and can induce synapses activation (45). And our previous study reported that NMDAR was activated during ECS stimulation (12), these data sufficiently suggest that neural system exerts an “activation state” during ECS treatment. Therefore, hypoexcitability-mediated threshold up-regulation after ECS will be revealed due to neural homeostasis. In addition, many studies have reported that general anesthetic agents (e.g. propofol, thiopental, etomidate and ketamine) exert a neuroprotective effect used in ECT/ECS (46–48). All general anesthetic agents provide loss of consciousness during the treatment, and ECS/ECT-induced neural activation is naturally suppressed, which may in turn support the idea that ECS/ECT-induced learning and memory deficits are caused by excitability-mediated metaplasticity.

Fan et al. and his colleagues found that a decrease in neural excitability was consistent with an up-regulation of *I*_h_ current after LTP induction in hippocampal slices (49). And the homeostatic changes were also confirmed in our series of studies. Our previous study found that ECS could raise the baseline fEPSP, which was identified as LTP-like synaptic changes (50). And in the present study, we found hippocampal neurons showed an increase in *I*_h_ current amplitude accompanied by a reduction in neural excitability and up-regulation of the LTD/LTP threshold after ECS, the *I*_h_ current inhibitor could reversed the changes. Essentially, the *I*_h_ current is a hyperpolarization-activated current comprised consisting of Na^+^ and K^+^. And it can modulate the decay rate and temporal summation of EPSPs and the depolarization process, which are responsible for the neural excitability (51). Furthermore, previous studies have reported that activation of NMADR, Ca^2+^ influx through NMADR and activation of the downstream protein CaMKII are critical for the up-regulation of *I*_h_ current (52). All these results support that neural activation seems to be a trigger for the adjustment of *I*_h_ current. Narayanan et al. even suggested that *I*_h_ current confer the ability for neurons to self-adjust their response properties to match the stimulus and can be used as a measure of recent neural activity (52). In the model of ECS, the following mechanic chain is distinct: neural system is activated during ECS treatment, resulting in subsequent *I*_h_ current increase, accompanied by a reduction in neuronal excitability, followed by metaplastic changes with LTD/LTP threshold up-regulation. This contributes to ECS-induced learning and memory dysfunction. This mechanism will provide a valuable version for the neuroprotection during ECS treatment: the “trigger” (the activation state) should not be sparked excessively, and general anesthetics have the ability to suppress the activation. And subsequent changes should be another target, neuroprotection has been identified by the *I*_h_ current inhibitor (such as ZD7288). After all, the aforementioned data are relation to functional synaptic plasticity. Changes in structural synaptic plasticity can also influence learning and memory function. Our team found that the number of synapses were increased after ECS, however, the ratio of perforated synapses was decreased and postsynaptic density was attenuated (unpublished data). Taken as a whole, changes in functional and structural synaptic plasticity should be involved in ECS-induced learning and memory impairment. However, more evidence should be probed to verify this contention.

There are several limitations to the present study. Firstly, we have proved the vital role of *I*_h_ current in the ECS-induced learning and memory impairment and speculated the neural activation may be a trigger for the subsequent changes in *I*_h_ current. However, we have not investigated the molecular mechanisms underlying the changes in *I*_h_ current. Secondly, neuronal excitability can be influenced by active neuronal membrane properties, which is closely correlated with changes in voltage-gated ion channels. *I*_h_ current is just a hyperpolarization-activated cationic current. To illustrate the relationship between *I*_h_ current and neuronal excitability preferably, only active neuronal membrane properties, not passive neuronal membrane properties were explored in this study.

## Conclusion

This study demonstrates that ECS-induced learning and memory impairment in depressed rats is caused by an increase in *I*_h_ current, followed by the changes in neuronal excitability-mediated metaplasticity with LTD/LTP threshold up-regulation. And the *I*_h_ current inhibitor ZD7288 can reversed changes induced by ECS and exerts a neuroprotective effect for ECS treatment.

## Data availability statement

The raw data supporting the conclusions of this article will be made available by the authors, without undue reservation.

## Ethics statement

The animal study was approved by the Ethical Committee of the First Affiliated Hospital of Chongqing Medical University. The study was conducted in accordance with the local legislation and institutional requirements.

## Author contributions

LR: Writing – original draft, Writing – review & editing, Conceptualization, Data curation. JY: Data curation, Writing – review & editing, Software. HC: Software, Writing – review & editing, Methodology. JL: Writing – review & editing, Resources. FL: Data curation, Investigation, Methodology, Writing – original draft. SM: Writing – original draft, Writing – review & editing.

## Supplementary material

Supplementary Figure 1 Animal experiment design schedule. CMUS, chronic unpredictable mild stress, SPT, sucrose preference test; MWM, Morris water maze.
