# Supplementary material for: Alteration of hyperpolarization-activated cation current-mediated metaplasticity contributes to electroconvulsive shock-induced learning and memory impairment in depressed rats
Source: Front Psychiatry. 2024 Jun 7;15:1365119. doi: 10.3389/fpsyt.2024.1365119 (PMC11190359; doi:10.3389/fpsyt.2024.1365119)
Supplement: Supplementary file 1 [file Table_1.docx]

Supplemental Table 1 Number of rats/slices/neurons in different experimental projects

| Item | No. of rats | No. of slices | No. of neurons |
| --- | --- | --- | --- |
| sucrose preference test |  |  |  |
| each group | 6 | / | / |
| Morris water maze |  |  |  |
| each group | 6 | / | / |
| LTD/LTP threshold |  |  |  |
| Group Sham |  |  |  |
| 10 HZ | 3 | 8 | / |
| 20 Hz | 3 | 8 | / |
| 40 Hz | 3 | 8 | / |
| Group ECS |  |  |  |
| 50 HZ | 3 | 8 | / |
| 80 HZ | 3 | 8 | / |
| Group ECS+ZD7288 |  |  |  |
| 40 HZ | 4 | 11 | / |
| 50 HZ | 3 | 9 | / |
| Group Sham+ZD7288 |  |  |  |
| 10 HZ | 3 | 10 | / |
| 20 Hz | 3 | 10 | / |
| 40 Hz | 4 | 11 | / |
| Spontaneous/evoked AP |  |  |  |
| Group Sham | 6 | 14 | 16 |
| Group ECS | 4 | 12 | 15 |
| Group ECS+ZD7288 | 5 | 14 | 18 |
| Group Sham+ZD7288 | 5 | 15 | 17 |
| *I*_h_ current |  |  |  |
| Group Sham | 5 | 16 | 20 |
| Group ECS | 5 | 12 | 15 |
| Group ECS+ZD7288 | 5 | 15 | 17 |
| Group Sham+ZD7288 | 4 | 12 | 16 |
