# Supplementary figures and images for: Alteration of hyperpolarization-activated cation current-mediated metaplasticity contributes to electroconvulsive shock-induced learning and memory impairment in depressed rats
Source: Front Psychiatry. 2024 Jun 7;15:1365119. doi: 10.3389/fpsyt.2024.1365119 (PMC11190359; doi:10.3389/fpsyt.2024.1365119)

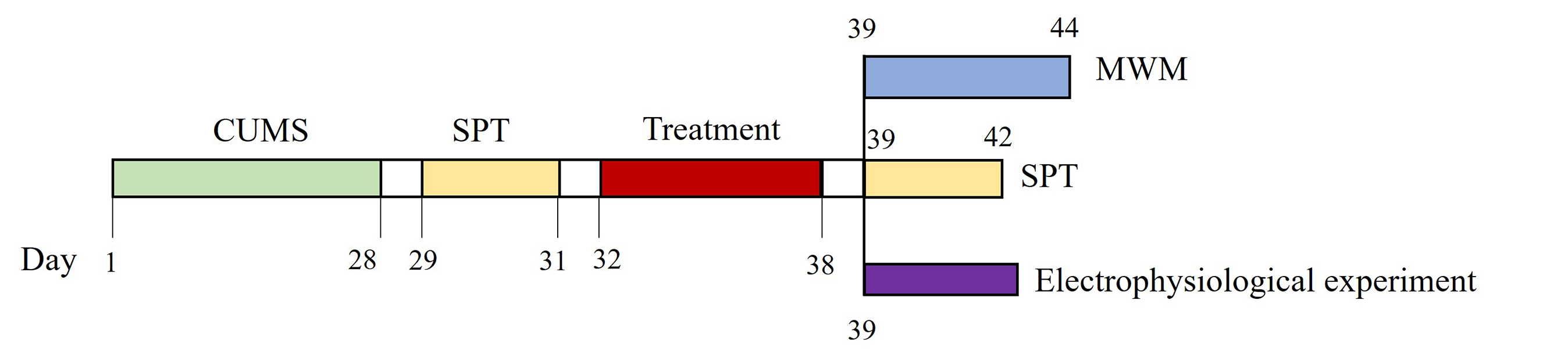

Supplement: Supplementary Figure 1 — Animal experiment design schedule. CMUS, chronic unpredictable mild stress, SPT, sucrose preference test; MWM, Morris water maze. [file Image_1.tif]
